# Supplementary material for: A systematic review to identify research gaps in studies modeling MenB vaccinations against Neisseria infections
Source: PLoS One. 2025 Jan 2;20(1):e0316184. doi: 10.1371/journal.pone.0316184 (PMC11694989; doi:10.1371/journal.pone.0316184)
Supplement: S1 File — (DOCX) [file pone.0316184.s001.docx]

# Supplementary Information


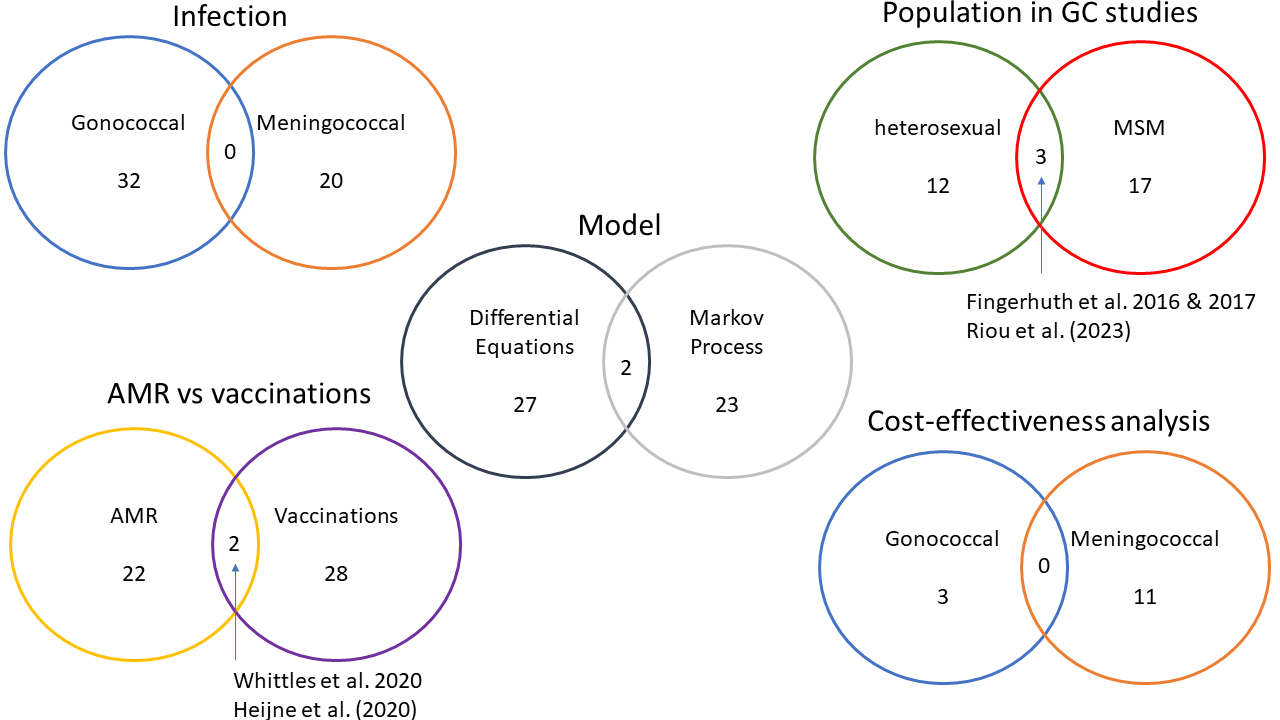


Figure S1: Main categorizations of included studies. Note that 1) none of the studies has looked into the impact of a MenB vaccine on both GC and MC, and 2) none of the GC studies modelled a spill over of infections from the MSM to the heterosexual community or vice versa.

Table S1: studies which included meningococcal infection and health economic analysis

| **Author** | **Question** | **Strategies** | **Dose schedule** | **Perspective** | **Time horizon** | **Measure of benefit** | **Results** | **Discounting** | **Vaccine cost / price** | **Vaccination or infection sequalae** | **Results sensitive to** | **Cost-effective price in base-case** |
| --- | --- | --- | --- | --- | --- | --- | --- | --- | --- | --- | --- | --- |
| (Beck et al., 2021) | MC: To estimate the broad impact and cost-effectiveness of Serogroup B Invasive Meningococcal Disease for infants in England | Vaccination versus no vaccination  **Scenarios / strategies:**  Disease burden categories (n=5) added incrementally.  1. Long-term sequelae  2 Spillover effects on family, and network/caregivers  3a Productivity losses  3b Productivity losses for spillover, e.g. family home.  3c Special educational needs  3d Formal long-term caregiving  3e Public health response  3f Litigation costs  4a Disease severity / adjustment factor  5 Long term impact of infection.  All burden categories;  Disease incidence high  Disease incidence low  Carriage effect included  No cross-protection  Vaccine effectiveness  Adverse events  Productivity losses  Discount rate | 2+1 vaccination schedule where priming doses are administered at 2 and 4 months and a booster dose is administered at 12 months of age. | *Societal perspective* | 100 years | QALYs productivity loss. | £360 595 per QALY taking a narrower perspective. £18,645 per QALY taking into account all factors | 3.5% for costs and benefits. Varied in scenario analysis | £75 per dose  £9.76 per dose | Impact on costs and utilities modelled separately:  Short-term infection sequelae Long-term infection sequelae: Amputation  Skin scarring  Renal dysfunction/ failure/ insufficiency  Neurological sequelae  Blindness/severe visual impairment  Hearing loss severe/ profound bilateral/deafness (cochlear implant)  Hearing loss moderate bilateral  Hearing loss unilateral/ hearing impairment  Epilepsy/seizures  Severe neurological disorders  Speech or communication problems  Mental retardation/ low IQ  Motor deficits  Psychological and behavioral sequelae  Depression  Anxiety  Separation anxiety  ADHD | MenB Incidence  Quality of life adjustment factor  Probability of long-term psychological and behavioural sequelae | Not reported |
| (Bos, et al., 2006) | MC: To estimate the epidemiological and economic impact of a combined 9-valent B and pneumococcal vaccine for all new-borns in The Netherlands | Vaccination versus no vaccination  **Scenarios / strategies:**  (i) no cross-reactivity (vaccine coverage 50%);  (ii) cross-reactivity based on Vermont et al (vaccine coverage 67%, base case scenario); and  (iii) maximum cross-reactivity (vaccine coverage of 84%) | 2, 3, 4 and 11 months | *Societal perspective* | Lifetime | QALYS | €17 700 per QALY. | 4% for cost and benefits | €40 per dose  Administration cost  €6.20 | **Infection:**  Neurological; physical;  hearing loss; invasive pneumonia and two health states from the EQ-5D (212111, 112112)  **Vaccination:** None | Changes in incidence;  vaccine price;  duration of protective efficacy. |  |
| (Breton, et al., 2020) | MC: To estimate the expected reduction of Serogroup B meningococci invasive meningococcal disease cases in the 30 years following introduction of routine age targeted vaccination in the Canadian population. | Vaccination versus no vaccination  **Scenarios / strategies:**  **(1)** age 14, along with existing school-based programs, with 75% uptake;  **(2)** age 17 with 75% uptake, assuming school vaccination; and  **(3)** age 17 with 30% uptake, assuming vaccination outside of school. | 2 dose schedule | *Societal perspective* | 30-years | Cases averted;  QALYs | $976,000 per QALY, $685,000 per QALY, and $490,000 per QALY. | 3% for both costs and benefits | $156.44 for the 2-dose series  Administration cost of $10.10 per dose | **Infection:**  Impact using direct medical costs, productivity losses and disutilities:  Scarring  Amputation  Paralysis  Seizure / epilepsy  Hearing loss  Neurologic sequelae  Renal failure  Vaccination:  One off vaccine disutility. | Tornado diagram suggestions the following 4 top factors:  MenB incidence  Vaccination costs  Vaccine uptake  Vaccine efficacy against carriage | $11 per dose (cost per QALY $135,000 per QALY) |
| (Christensen, et al., 2013) | MC: Predict the potential impact of introducing a new vaccine in England, with the capacity to protect against serogroup B meningococcal disease | **Vaccination versus no vaccination**  **A:** Infant: 2,3,4 + 12 months of age  **B:** Infant: 2,4,6 + 12 months of age  **C:** Infant: 2,3,4 months of age  **D:** Infant: 2,3,4 + 12 months of age  **E:** Infant: 2,3,4 + 12 months of age  **F:** Adolescent: 0, 2, 6 schedule  **G:** Adolescent: 0, 2, 6 schedule | Various different, as strategies. | NHS and personal and social services | 100 years | cases averted; deaths averted; QALYs | £162,800 per QALY | 3.5% for the first 30 years;  3.0% in years 31–75 and 2.5% in years 76–99 all for cost and benefits | Assumed £40 per dose  Cost of administration at school, per dose £5.6 | **Infection:**  Minor sequalae / major sequalae impacting on QoL (-0.2) and annual cost (£500, £10,000)  **Vaccination:** None | Vaccine profile;  disease incidence;  case fatality; sequelae, including quality of life losses and costs of care. | £9 per dose |
| (Christensen, et al., 2014) | MC: Predict the potential impact of introducing a new vaccine in England, with the capacity to protect against serogroup B meningococcal disease | Vaccination versus no vaccination  **Scenarios / strategies:**  **Routine Infant**  2, 3, 4, and 12 months;  2, 3, 4, and 12 months;  2, 4, and 12 months;  2, 3, 4, and 12 months;  **Routine adolescent**  13 years‡  13 years  **Routine infant and adolescent**  2, 3, 4, and 12 months;  13 years | Various different, as strategies. | Societal perspective | 100 years | cases averted; QALYs | £221,000 per QALY | 3.5% for cost and benefits | List price of Bexsero.  Administration cost £7.50 | **Infection:**  Quality of life losses during the acute disease episode, deriving estimates from PHE study using EQ-5DY in children up to a year after the illness.  Long term reductions in quality of life for survivors with sequelae using data from the MOSAIC study.  Some cases were assumed to result in claims against the NHS, attracting legal costs and damages not related to quality of life. Where included, QoL losses were ignored to avoid double counting.  **Vaccination:** None | Vaccine profile;  Impact of disease on the person affected as well as family and network;  QoL adjustment factor | £3 per dose |
| (Christensen, et al., 2016) | MC: Predict the potential impact of introducing a new vaccine in England, with the capacity to protect against serogroup B meningococcal disease | Vaccination versus no vaccination  **Scenarios / strategies:**  **Routine infant**  2, 3, 4, and 12 months  2, 3, 4, and 12 months  2, 3, 4, and 12 months  2, 4, 6 + 12 months  2, 4, 6 + 12 months  6, 8, 12 months  6, 8, 12 months  **Routine adolescent**  12 years  12 years (0, 2 schedule)  **Routine infant and adolescent**  2, 3, 4, and 12 months; 12 years;  6, 8 and 12 months; 12 years | Various different, as strategies. | Payer perspective | 100 years | cases averted; deaths averted; QALYs | €2,015,300 per QALY | 3% for cost and benefits | €96.96 per dose  Administration cost €6.50 | **Infection:**  Cost of support care for those with mild / severe sequalae (annual).  QoL loss for survivors with and without sequalae over first year (not differentiated by severity).  Ongoing QoL for survivors with sequalae, not differentiated by age.  **Vaccination:**  Costs of hospitalisations for severe fever and anaphylaxis as possible adverse events following vaccination;  did not include possible quality of life losses associated with adverse events | Results were sensitive to: disease incidence.  Results were robust (being not cost-effective) to favorable vaccine profile assumptions; herd effects; use of societal perspective. | <1 per dose |
| (Christensen & Trotter, 2017) | MC: investigate the cost-effectiveness of different catch-up options, focusing not on children under 11 years, but on the birth cohorts after infancy who experience the greatest disease burden, i.e. 1, 2 and 3–4 year olds | Vaccination with catch-up versus no catch-up  **Scenarios / strategies:**  2,4 + 12 months + CU in 1 y;  2,4 + 12 months + CU in 1–2 y;  2,4 + 12 months + CU in 1–4 y | Various different, as strategies. | NHS and personal and social services | 100 years | QALYs | £273,400 per QALY | 3.5% for cost and benefits  1.5% for costs and benefits | List price of Bexsero.  Administration cost £9.80 | **Infection:**  Quality of life losses during the acute disease episode, deriving estimates from PHE study using EQ-5DY in children up to a year after the illness.  Long term reductions in quality of life for survivors with sequelae using data from the MOSAIC study.  Some cases were assumed to result in claims against the NHS, attracting legal costs and damages not related to quality of life. Where included, QoL losses were ignored to avoid double counting.  **Vaccination:** None | Reducing discount rate for costs and benefits improved cost-effectiveness.  Disease incidence over time.  Vaccine strain coverage and herd effects reduces cost-effectiveness.  Family and network QALYs improves cost-effectiveness. | £13 per dose |
| (Gasparini, et al., 2016) | MC: cost-effectiveness analysis (CEA) on the possible use of BexseroÒ in the Italian epidemiological scenario | Vaccination versus no vaccination  **Scenarios / strategies:**  1: Perspective: social; cost of death 0; number of vaccine doses: 5; disease incidence: official data  2: Perspective: social; cost of death SHC; number of vaccine doses: 5; disease incidence: official data  3. Perspective: social; cost of death WTP; number of vaccine doses: 5; disease incidence: official data  4. Perspective: NHS; cost of death WTP; number of vaccine doses: 5; disease incidence: official data  5. Perspective: social; cost of death 0; number of vaccine doses: 5; disease incidence: estimated data  6. Perspective: social; cost of death SHC; number of vaccine doses: 5; disease incidence: estimated data  7. Perspective: social; cost of death WTP; number of vaccine doses: 5; disease incidence: estimated data  8. Perspective: NHS; cost of death WTP; number of vaccine doses: 5; disease incidence: estimated | 2, 4, 6 and 12 months of age with a booster dose at 11 years. | Societal and health care provider perspective | Not reported | Death, survival without sequelae, and survival with long-term sequelae;  QALYs | €109,762 per QALY for scenario 1;  37,827 per QALY for scenario 8.  Not fully incremental analysis. | 3% for both costs and benefits | €200 (4 doses)  Administration cost per dose €5.80 | **Infection:**  Impact on costs and QoL of:  Amputation with substantial  Depression  Motor deficits  Blindness  Epilepsy or Seizure  Severe Neurological disability  Mental retardation (cognitive problems)  Hearing loss requiring cochlear implantation  Moderate/severe bilateral hearing loss  Moderate unilateral hearing loss  Skin necrosis  Scars  Severe speech or communication problems  Renal failure  Chronic migraine  **Vaccination:**  None | Tornado diagram showing incidence of disease had the largest impact on the ICER.  Probability of sequalae also had a large impact. | Not reported |
| (Lecocq, et al., 2016) | MC: To conduct an economic evaluation to support the haut Conseil de la Santé Publique, (HCSP) make its recommendation regarding the potential integration of Bexsero® into the immunization schedule | Vaccination versus no vaccination  **Scenarios / strategies:**  Infant strategy (A): primary series at 3, 5 and 6 months and a booster dose at 13 months  Toddler strategy (B): 2 primary doses at 13 and 15 months with a booster dose at 27 months  Adolescent strategy (C): 2 doses one month apart in adolescents at 15 years  Booster strategy (D): booster dose at 15 years old and a catch-up for 15 years old subjects (2 doses one month apart) during the first 15 years of the program were added to the infant  Booster strategy (E): booster dose at 15 years old and a catch-up for 15 years old subjects (2 doses one month apart) during the first 15 years of the program were added to the toddler. | Various different, as strategies. | Restricted societal perspective with direct costs only | 100 years | QALYs | Infant vaccination €380,973 per QALY  Adolescent vaccination €135,902 per QALY  Infant vaccination with a late booster and catch-up €188,511 per QALY gained. | 4% for the first 30 years with a progressive disease to 2% thereafter for both costs and benefits. | €40 per dose  Administration cost €23.61 – €27.82 | **Infection:**  Impact on QoL:  Severe hearing loss  Mild hearing loss  Blindness  Full IQ less than 85  Epilepsy  ADHD  Amputation  Impact on costs:  Handicap cost per year  **Vaccination:**  Adverse effect cost per dose (bundled together):  Fever;  febrile seizure;  Kawasaki disease  juvenile arthritis | Tornado diagram consistently showing these to be the most sensitive:  Discount rate;  Vaccine cost;  Incidence;  Waning rate of protection  Although results were robust as none were cost-effective | Not reported |
| (Pouwels, et al., 2013) | MC: The cost-effectiveness of vaccine implementation strategies for meningococcal B in the Netherlands at differing levels of disease incidence. | **Scenarios / strategies:**  1: 2, 3, 4, 11 months  2: 2, 3, 4, 11 months + 12 years  3: 12 + 14 months  12 + 14 months + 12 years  **Base-case disease incidence**: 1.07 per 100k  **1990 – 1993 disease incidence:** 3.46 per 100k |  | Societal | 100 years | QALYs | €243,778 per QALY | 4% and 1.5% for costs and benefits | €40 per dose  Administration cost €6.81 | **Infection:**  **Impact on costs:**  Cochlear  Scars  Hospitalization for scars  Amputations  Amputation hospitalization  Special education  Institution care  **Impact on QoL:**  Hearing loss;  Motor deficits;  Scars;  Amputations; Neurological sequelae (not specified) | Tornado diagram shows:  vaccine effectiveness; total vaccination costs;  case-fatality rate (CFR);  proportion of cases with sequelae;  incidence of MenB disease;  duration of protection provided by the vaccine | £13 per dose |
| (Scholz, et al., 2022) | MC: To assess the cost-effectiveness of Routine Infant 4CMenB Vaccination in Germany to Prevent Serogroup B Invasive Meningococcal Disease | Vaccination versus no vaccination  **Scenarios / strategies:**  Quality of life adjustment factor (QAF) of 3 applied to QALY loss in cases with long-term sequelae  - Incidence increased by 16.7% to account for potential underreporting  - Standard operating procedure (SOP) scenario developed according to STIKO SOP, with 3% discount rates for costs and QALYs  - Base case scenario assumes no carriage effect of 4CMenB  - Carriage scenario assesses the potential effectiveness of 4CMenB in preventing acquisition of Nm carriage and the impact of herd protection  - High and low incidence scenarios conducted with incidence multiplied by 3 and 0.5, respectively | 2 to 1 dose schedule  at 2, 4 and 12 months of age | Societal | 100 years | IMD cases averted, MenB cases averted, QALYs gained. | €188,762 per QALY | 1% for costs and 1% benefits | Vaccine price of €97.06  Administration cost €7.60 | Same list of sequelae as Beck 2021 with impact on both utilities and costs. Different cost values. | Incorporation of additional ‘value’ factors heavily impacted upon ICER. Narrower perspective is €817,000 per QALY. | Not calculated. |

*assumed £30,000 per QALY unless otherwise stated

MC: meningococcal, GC: gonococcal, QALY: quality-adjusted life year; VOD: Vaccine on diagnosis, VAR: Vaccine at risk, VoA: Vaccine on attendance; ICER: incremental cost-effectiveness ratio.

Table S2: studies which included gonococcal infection and health economic analysis

| **Author** | **Question** | **Strategies** | **Dose schedule** | **Perspective** | **Time horizon** | **Measure of benefit** | **Results** | **Discounting** | **Vaccine cost / price** | **Vaccination or infection sequalae** | **Results sensitive to** | **Cost-effective price in base-case** |
| --- | --- | --- | --- | --- | --- | --- | --- | --- | --- | --- | --- | --- |
| (Régnier & Huels, 2014) | GC: To assess the impact of a vaccination campaign with the 4CMenB vaccine on gonorrhoea outcomes in the USA | Vaccination in adolescents versus no vaccination (treatment with antibiotics for infection)  **Scenarios / strategies:**  Differing levels of vaccine effectiveness: 10%, 20%, 30%, 40%, 50%  Different levels of antibiotics efficacy: 0%, 25%, 50%, 75%, 97% | Two dose schedule at adolescence. | Societal | Lifetime | QALYs | Economically justifiable price calculated | 3% for costs and benefits | A function of the results | **Infection:**  Women: PID; Ectopic pregnancy; Chronic pelvic pain; Infertility  Men: Urethritis; Epididymitis; Incremental HIV infection(?)  **Vaccination:**  None | Results / drivers of value are in the following order:  Sequalae / symptoms  HIV  Productivity  Treatment / diagnosis. | $26.10 at a threshold of £75,000 per QALY (20% effectiveness and 97% effective antibiotics) |
| (Whittles, et al., 2022) | GC: Public health impact and cost-effectiveness of gonorrhoea vaccination for MSM in England. | Vaccination scenarios versus no vaccine:  **Scenarios / strategies:**  - Vaccination before entry (VbE): This strategy involves vaccinating adolescents in schools before they become sexually active.  - Vaccination on diagnosis (VoD): This strategy involves vaccinating men who have sex with men (MSM) in sexual health clinics when they are diagnosed with gonorrhoea.  - Vaccination on attendance (VoA): This strategy involves vaccinating MSM in sexual health clinics when they attend the clinic, regardless of whether or not they are diagnosed with gonorrhoea.  - Vaccination according to risk (VaR): This strategy involves vaccinating individuals based on their current infection with gonorrhoea or their self-reported high number of sexual partners. It is a hybrid approach, using VoD for those with low activity and VoA for those with high activity. | 2 dose schedule with additional booster for waning protection. | Sexual health clinics | 10 and 20-years | Cases averted, value per dose. QALYs | £18 per dose:  VoD and VaR dominate comparator. VoA has an ICER <£10,000 per QALY.  £85 per dose:  VOD ~£12,000 per QALY  VAR ~ £15,000 per QALY  VOA ~ £80,000  VOD is externally dominated. | 3.5% for costs and benefits | List price of £75 per dose  Administration cost per dose  £10 | **Infection:**  None  **Vaccination:**  None | Vaccine efficacy  Duration of protection Targeting strategy | Cost saving combined with cases avoided. |
| (Xiridou, et al., 2016) | GC: Cost-Effectiveness of Dual Antimicrobial Therapy for Gonococcal Infections Among Men Who Have Sex With Men in the Netherlands | Antibiotics ceftriaxone and azithromycin compared with monotherapy of ceftriaxone | N/A | Healthcare provider | 10, 20, 30, 40, 50, 60  (base-case not identified) | QALYs | N/A | 4% for costs and 1.5% for benefits | N/A | **Infection:**  None  **Vaccination:**  None | N/A | N/A* |

*Table S3: Journal articles and grey literature matching our inclusion and exclusion criteria, by year.*

| **Year** | **Articles** | **Conference Presentations** | **Preprints** | **Theses** |
| --- | --- | --- | --- | --- |
| 1990 | 1 |  |  |  |
| 2002 | 1 |  |  |  |
| 2006 | 2 |  |  |  |
| 2012 | 2 |  |  |  |
| 2013 | 2 |  |  |  |
| 2014 | 4 | 2 |  |  |
| 2015 | 4 | 2 |  | 1 |
| 2016 | 7 |  |  |  |
| 2017 | 5 | 7 |  |  |
| 2018 | 1 |  |  | 1 |
| 2019 | 2 | 8 |  |  |
| 2020 | 5 | 5 | 2 |  |
| 2021 | 3 |  |  |  |
| 2022 | 6 |  |  | 1 |
| 2023 | 3 | 2 | 1 |  |
