# Supplementary material for: A systematic review to identify research gaps in studies modeling MenB vaccinations against Neisseria infections
Source: PLoS One. 2025 Jan 2;20(1):e0316184. doi: 10.1371/journal.pone.0316184 (PMC11694989; doi:10.1371/journal.pone.0316184)
Supplement: S5 File — (DOCX) [file pone.0316184.s005.docx]

**Could vaccinating at risk populations with Meningococcal B vaccine reduce incidence and antimicrobial resistance in gonococcal (GC) infections in the UK? A systematic review protocol to develop a transmission model of GC and MenB infection for the UK**

**Authors:** The University of Manchester, UKHSA

**Contact details for further information:** ian.hall@manchester.ac.uk

**Type and method of review:** systematic review, descriptive and narrative synthesis

**Start date:** 01/10/2021

**Completion date:**

**Funding:** Wellcome Trust

1. **Background**

Antimicrobial resistance (AMR) threatens public health and individual patient care. Gonococcal (GC) infection incidence has been increasing year on year for the last decade in the UK.^1,2^ AMR in GC infection is relatively low in the UK, but it has been increasing too.^1^ More worryingly, multi-drug resistant GC (MDR-GC) and XDR-GC are fast emerging elsewhere, and the first two cases of MDR-GC have been diagnosed and acquired in the UK. ^3^

*Neisseria gonorrhoeae* and *Neisseria meningitidis* are closely related bacteria that cause a significant global burden of disease. Control of gonorrhoea is becoming increasingly difficult due to widespread antibiotic resistance. While vaccines are routinely used for *N. meningitidis*, no vaccine is licensed for *N. gonorrhoeae*. A recent study in New Zealand and Cuba where outer membrane vesicle (OMV) meningococcal B (MenB) vaccine was given to adolescents was reported with 30% reduction in incidence rates of GC in those vaccinated, as the vaccine potentially offers some cross protection.^4,5,6,7,9^

The UK, since 2015 have offered the MenB vaccine as part of the national infant immunisation schedule.^6^ This vaccine is Bexsero, and one component is the NZ OMV. Cost-effectiveness of the MenB vaccine against meningococcal disease in adolescents in the UK is borderline given the relatively low incidence of *N. meningitidis* group B infections and the cost of the vaccine; hence immunisation has been targeted in the UK to infants.^8^ For this to have any noticeable effect on the incidence of GC infections it will take another 20 years.

We propose to model the cost-effectiveness of vaccinating groups at risk of GC against MenB and comparing this with offering vaccination to adolescents and continuing with childhood vaccination. We will measure the reduction in MenB and GC incidence and AMR.

1. ***Aim***

To explore whether targeted immunisation with Meningococcal B vaccine to populations at risk of gonococcal infection will reduce incidence and resistance in gonorrhoea.

1. ***Research question***

This review seeks to answer the question “what evidence exists at present for whether targeted immunisation with Meningococcal B vaccine to populations at risk of gonococcal infection reduce incidence and antimicrobial resistance in gonorrhoea?”

1. ***Specific objectives***

- Inform development of a transmission model of GC and MenB infection for the UK
- Investigate the cost-effectiveness of MenB vaccine in infants, adolescents, and targeted at-risk populations in reducing MenB and GC infection incidence and AMR.
- Investigate the potential impact in areas of low, medium, and high incidence of GC infection and low- and high-level AMR in GC.

1. ***Criteria for studies inclusion (PICOS)***

| 1. Participants or Population | This review will consider all studies that involve   - All persons eligible for MenB vaccination |
| --- | --- |
| 1. Interventions | Interventions of interest included those related to the following:   - Effectiveness and/or efficacy of MenB Vaccine; - Continuation of existing vaccination programmes; - Screening systems; - Assessment strategies of medication; - Intervention programmes; - Specific clinical interventions |
| 1. Comparisons | Infection levels and AMR in targeted groups at greater risk of gonococcal infections with and without vaccination |
| 1. Outcome of Interest | - A transmission model at population scale of GC and MenB infection for the UK - Cost-effective vaccination strategies to reduce MenB and GC infection incidence and AMR. - Simulated planned activities for Vaccine strategies using the transmission dynamic model of GC and MenB infection and vaccination |
| 1. Study designs | Modelling using direct or indirect measurement methods to evaluating the effectiveness or efficacy of interventions/strategies relating to gonococcal or meningococcal infections, and the impact on AMR in this infection. |

1. ***Search methods***

| Electronic databases | Journal publications:   - Medline, Embase (both via Ovid) - PubMed - Scopus   Preprints:   - medRxiv - OSF Preprints (incl. aRxiv, bioRxiv…)   Grey literature:   - base-search.net - British Library - OpenGrey |
| --- | --- |
| Other methods used for identifying relevant research. | 1. Reference checking and hand searching of these 2. Terms identified, and the synonyms used by respective databases, will be used in an extensive search of the literature. 3. Reference lists and bibliographies of the articles collected from those identified. |

1. ***Study selection***

***The inclusion criteria:***

- Mathematical Model (with transmission or fundamental mechanisms captured in model) in the title or abstract.
- Title and abstract must cover either or both of the following infections (diseases) and synonyms:
  - Gonococcal infection (gonorrhoea)
  - Meningococcal infection, serogroup B (bacterial meningitis)
- Title and abstract should contain at least one of the following two subject matters
  - AMR
  - Vaccination with MenB or Bexsero

***The exclusion criteria:***

- All non-primary studies, conference talks and studies not available in English will be excluded. Only primary studies publishing gonococcal infections are of interest.
- Papers reporting genomic sequencing, agricultural model, animal model, conventional statistical modelling or analysis, systematic literature reviews, and meta-analyses will be excluded from the review, unless they used or published de-novo data.
- Meningococcal conjugate vaccine that are not serogroup B.
- Screening the eligible articles for papers that do not answer the research questions of the study (for example, articles featuring the search terms but that are merely definitions, descriptions, or referred to for comparison, etc.).

1. ***Data synthesis***

All search results will be screened to eliminate duplicate entries. After deduplication, we will screen titles and abstracts for our inclusion and exclusion criteria as defined above to reduce our list further. We will end up with a list of studies that address our objectives (included studies). After data collection, a qualitative synthesis of the included studies will be used to organise existing GC modelling studies. An extraction form will be developed using Excel or any other suitable program, based on the following categories: study title, infectious disease system, model type, model formulation/class, transmission route, methodology, validation technique, intervention target, and type of data used**.** Additional rounds of data extraction with subgroups of the included studies will be performed, as necessary.

Two or more appropriately qualified persons will extract and enter data independently from each included study. Inconsistencies in data extraction or data entry will be resolved by consensus. If there is no agreement, an independent reviewer will intervene to arrive at a final decision. Studies might be excluded at the data entry stage if it becomes apparent that inclusion criteria are not met or there is not enough information in the documents to extract the required data.

A descriptive analysis of the data generated from the systematic search, in line with the study protocol, will be reported using flow charts (to illustrate included and excluded publications/registered trials) and tables (to present studies, models, and setting characteristics). The report will be written following the PRISMA Guidelines for reporting of systematic reviews and meta-analyses^10, 11^ to present the research methodology and findings.

**References**

1. Public Health England. Sexually transmitted infections and screening for chlamydia in England, 2018. Health protection Report. June 7, 2019. Link: <https://assets.publishing.service.gov.uk/government/uploads/system/uploads/attachment_data/file/806118/hpr1919_stis-ncsp_ann18.pdf>
2. Public Health England. Update on investigation of UK case of Neisseria gonorrhoeae with high-level resistance to azithromycin and resistance to ceftriaxone acquired abroad. Health Protection Report. April 20, 2018. Link: <https://assets.publishing.service.gov.uk/government/uploads/system/uploads/attachment_data/file/701185/hpr1418_MDRGC.pdf>
3. Whittles LK, White PJ, Paul J, Didelot X. Epidemiological Trends of Antibiotic Resistant Gonorrhoea in the United Kingdom. Antibiotics (Basel). 2018 Jul 13;7(3). pii: E60. doi: 10.3390/antibiotics7030060. Review.
4. Semchenko EA, Tan A, Borrow R, Seib KL. The serogroup B meningococcal vaccine Bexsero elicits antibodies to Neisseria gonorrhoeae. Clin Infect Dis. 2018 Dec14. doi: 10.1093/cid/ciy1061. [Epub ahead of print]
5. Petousis-Harris H, Paynter J, Morgan J, Saxton P, McArdle B, Goodyear-Smith F, Black S. Effectiveness of a group B outer membrane vesicle meningococcal vaccine against gonorrhoea in New Zealand: a retrospective case-control study. Lancet. 2017 Sep 30;390(10102):1603-1610. doi: 10.1016/S0140-6736(17)31449-6. Epub 2017 Jul 10.
6. Humbert MV, Christodoulides M. Immunization with recombinant truncated Neisseria meningitidis-Macrophage Infectivity Potentiator (rT-Nm-MIP) protein induces murine antibodies that are cross-reactive and bactericidal for Neisseria gonorrhoeae. Vaccine. 2018 Jun 22;36(27):3926-3936. doi: 10.1016/j.vaccine.2018.05.069. Epub 2018 May 24.
7. Acevedo R, Bai X, Borrow R, Caugant DA, Carlos J, Ceyhan M, Christensen H, Climent Y, De Wals P, Dinleyici EC, Echaniz-Aviles G, Hakawi A, Kamiya H, Karachaliou A, Lucidarme J, Meiring S, Mironov K, Sáfadi MAP, Shao Z, Smith V, Steffen R, Stenmark B, Taha MK, Trotter C, Vázquez JA, Zhu B. The Global Meningococcal Initiative meeting on prevention of meningococcal disease worldwide: Epidemiology, surveillance, hypervirulent strains, antibiotic resistance and high-risk populations. Expert Rev Vaccines. 2019 Jan;18(1):15-30.
8. Public Health England. MenB vaccination: introduction from September 2015. Correspondence. June 2, 2015. Link: <https://www.gov.uk/government/publications/menb-vaccination-introduction-from-1-september-2015>
9. Christensen H, Trotter CL, Hickman M, Edmunds WJ. Re-evaluating cost effectiveness of universal meningitis vaccination (Bexsero) in England: modelling study. BMJ. 2014 Oct 9;349:g5725. doi: 10.1136/bmj.g5725.
10. Liberati A, Altman DG, Tetzlaff J, Mulrow C, Gøtzsche PC, Ioannidis JP, Clarke M, Devereaux PJ, Kleijnen J, Moher D. 2009. The PRISMA statement for reporting systematic reviews and meta-analyses of studies that evaluate healthcare interventions: explanation and elaboration. BMJ. 2009; 339:b2700.
11. Moher D, Liberati A, Tetzlaff J, Altman DG. 2009. PRISMA Group. Preferred reporting items for systematic reviews and meta-analyses: the PRISMA statement. PLoS Med. 2009; 6:e1000097.

**Appendix 1: Data extraction form template**

|  | **Examples of Data Elements for Extraction** |
| --- | --- |
| **Study** | Author  year  Country |
| **Infectious disease system** | Neisseria gonorrhoeae  Meningococcal Infections  w/ or w/o Antimicrobial Resistance |
| **Model Type** | Population-based dynamic transmission model  Markov model  Economic model  Cohort model |
| **Model assumptions** | Assumptions made in model formulation by the study |
| **Model formulation/class** | Stochastic  Deterministic  Statistical  Hybrid  SIS  SVIR  SEIR  SIR  Age-structured  Multi-strain model |
| **Transmission Route** | Oral, Co-infection, Sexual |
| **Methodology used** | Differential equations (ODE or PDE)  Jump processes (stochastic)  Analytical  Neural networks |
| **Validation technique** | Model fitting  Model Calibration |
| **Intervention target** | Disease  AMR  Both |
| **Type of data used** | Clinical  Epidemiological  Experimental  Theoretical |
| **Cost-effectiveness** | Intervention costs  Cost–benefit analysis  Cost-effectiveness analysis  Cost–utility analysis etc. |

**Appendix 2: Search strings**

| **Ovid MEDLINE & EMBASE** |
| --- |
| **Mathematical Model term**  1 Model*, theoretical/  2 Model*, transmission/  3 Markov.mp.  4 (compartmental adj3 model*).mp.  5 micro simulation*.mp.  6 (mathematical adj3 model*).mp.  7 1 or 2 or 3 or 4 or 5 or 6  **Disease term**  8 Gonorrh*/  9 Neisseria/  10 Meningococcal/  11 Gonococcal/  12 8 or 9 or 10 or 11  **AMR & Vaccines**  13 Drug Resistan*.mp.  14 Antimicrobial Resistan*.mp.  15 Antibiotic resistan*.mp.  16 Bexsero.mp.  17 MenB.mp.  18 serogroup B vaccination.mp.  19 13 or 14 or 15 or 16 or 17 or 18  **Results**  20 7 and 12 and 19 |

**Appendix 3: SCOPUS & PubMed & pre-print/grey literature databases**

| **SCOPUS & PubMed & pre-print/grey literature databases** |
| --- |
| (“transmission model*” OR “theoretical model*” OR “mathematical model*” OR  “compartmental model*” OR Markov OR “micro simulation*”) AND   (Neisseria OR gonorrh* OR gonococcal OR meningococcal) AND   (Bexsero OR MenB OR “serogroup B” vaccination OR “drug resistan*” OR “antimicrobial resistan*” OR “antibiotic resistan*”) |

Scopus will be searched for Title-Abstract-Keywords.

base-search.net will be searched without the wildcard in phrase searches, using for example “transmission model” and “drug resistance”.
