# Supplementary material for: A systematic review to identify research gaps in studies modeling MenB vaccinations against Neisseria infections
Source: PLoS One. 2025 Jan 2;20(1):e0316184. doi: 10.1371/journal.pone.0316184 (PMC11694989; doi:10.1371/journal.pone.0316184)
Supplement: S6 File — (DOCX) [file pone.0316184.s006.docx]

**Could vaccinating at risk populations with Meningococcal B vaccine reduce incidence and antimicrobial resistance in gonococcal (GC) infections in the UK? A systematic review protocol to develop a transmission model of GC and MenB infection for the UK**

**Authors:**

The University of Manchester

**Contact details for further information:**

**Type and method of review:** systematic review, descriptive and narrative synthesis

**Start date:**

**Completion date:**

**Funding:**

1. **Background**

Antimicrobial resistance (AMR) threatens public health and individual patient care. Gonococcal (GC) infection incidence has been increasing year on year for the last decade in the UK.^1,2^ AMR in GC infection is relatively low in the UK, but it has been increasing too^1^. More worryingly, multi-drug resistant GC (MDR-GC) and XDR-GC are fast emerging elsewhere, and the first two cases of MDR-CG have been diagnosed and acquired in the UK ^3^.

*Neisseria gonorrhoeae* and *Neisseria meningitidis* are closely related bacteria that cause a significant global burden of disease. Control of gonorrhoea is becoming increasingly difficult due to widespread antibiotic resistance. While vaccines are routinely used for *N. meningitidis*, no vaccine is available for *N. gonorrhoeae*. A recent study in New Zealand and Cuba where outer membrane vesicle (OMV) meningococcal B (MenB) vaccine is given to adolescents was reported with 30% reduction in incidence rates of GC in those vaccinated, as the vaccine potentially offers some cross protection^4,5,6,7,9^.

In the UK, since 2015, we offer MenB vaccine^6^. Cost-effectiveness of the MenB vaccine against meningococcal disease in adolescents in the UK is borderline given the relatively low incidence of *N meningitidis* group B infections and the cost of the vaccine; hence immunisation has been targeted in the UK to infants^8^. For this to have any noticeable effect on the incidence of GC infections it will take another 20 years.

We proposed to model the cost-effectiveness of vaccinating groups at risk of GC against MenB and comparing this with offering vaccination to adolescents and continuing with childhood vaccination. We will measure the reduction in MenB and GC incidence, and AMR.

1. ***Aim***

To explore whether targeted immunisation with Meningococcal B vaccine to populations at risk of gonococcal infection will reduce resistance in gonorrhoea.

1. ***Research question***

This review seeks to establish, through the literature, whether targeted immunisation with Meningococcal B vaccine to populations at risk of gonococcal infection can reduce incidence and antimicrobial resistance in gonorrhoea?

1. ***Specific objectives***

- Develop a transmission model of GC and MenB infection for the UK
- Investigate the cost-effectiveness of MenB vaccine in infants, adolescents, and targeted at-risk populations in reducing MenB and GC infection incidence and AMR.
- Investigate the potential impact in areas of low, medium, and high incidence of GC infection and low- and high-level AMR in GC.

1. ***Criteria for including studies in the review***

| 1. Participants or Population | This review will consider all studies that involve   - Human subjects of any age who are sexually active - Vaccinating adolescents - Vaccination of targeted groups at greater risk of gonococcal infections (e.g. MSM, those who engage in commercial sex) |
| --- | --- |
| 1. Interventions | Interventions of interest included those related to the following:   - Effectiveness and/or efficacy of MenB Vaccine; - Continue vaccination programme of infants; - Screening systems; - Assessment strategies of medication; - Intervention programmes; - Specific clinical interventions |
| 1. Comparisons | Targeted groups at greater risk of gonococcal infections (e.g. Sexually active individual, MSM, those who engage in commercial sex) |
| 1. Outcome of Interest | - A transmission model at population scale and within an individual of GC and MenB infection for the UK - Cost-effective vaccination strategies to reduce MenB and GC infection incidence and AMR. - Simulated planned activities for Vaccine strategies using the transmission dynamic model of GC and MenB infection and vaccination |
| 1. Study designs | Empirical study using direct or indirect measurement methods to evaluating the effectiveness or efficacy of interventions/strategies relating to gonococcal infections, and the impact on AMR in this infection. |

1. ***Search methods***

| Electronic databases | Medline, Embase (both via Ovid) and Scopus |
| --- | --- |
| Other methods used for identifying relevant research. | 1. Reference checking and hand searching of these 2. Terms identified, and the synonyms used by respective databases, will be used in an extensive search of the literature. 3. Reference lists and bibliographies of the articles collected from those identified. |

1. ***Study selection***

***The inclusion criteria:***

- Mathematical model in the title or abstract, Mathematical model of AMR or gonorrhoea. Transmission models at population level, Vaccine or Bexsero and Meningococcal (Sero groups)
- English language or available translation.
- References for gonococcal infections inputs cited by included studies will be assessed as citation snowballing exercise.

***The exclusion criteria:***

- All non-primary studies, conference abstracts and studies not available in English will be excluded. Only primary studies publishing gonococcal infections are of interest.
- Papers reporting agent-based model, genomic sequencing, agricultural model, animal model, submissions, systematic literature reviews, and meta-analyses will be excluded from the review, unless they used or published de-novo data.

## ***Data collection***

Data and estimates for modelling transmission gonococcal infections and vaccination will come from:

- Gonococcal infections – [GUMCAD](https://www.gov.uk/guidance/gumcad-sti-surveillance-system) (PHE)
- AMR in CG – [GRASP](https://www.gov.uk/government/publications/gonococcal-resistance-to-antimicrobials-surveillance-programme-grasp-protocol) (PHE)
- AMR in MenB – Meningococcal reference Laboratory (PHE)
- Meningococcal B infections – Meningitis surveillance (PHE)
- Meningococcal B mortality – HPzone (PHE) or Mortality register (ONS – England & Wales)
- Healthcare costs – HES (access through PHE)
- Sexual behaviours and at-risk groups in the UK population – NATSAL3
- Vaccine uptake – [ImmForm](https://www.gov.uk/government/collections/immunisation) (DHSC, access through PHE)

1. ***Data synthesis***

Data collated from individual studies will not be synthesised but summarised using a descriptive analysis supported by a narrative of the methods used to generate utility values for each specified health state.

Studies will be categorised according to the health state(s) and the study population for which the utility values were elicited.

**References**

1. Public Health England. Sexually transmitted infections and screening for chlamydia in England, 2018. Health protection Report. June 7, 2019. Link: <https://assets.publishing.service.gov.uk/government/uploads/system/uploads/attachment_data/file/806118/hpr1919_stis-ncsp_ann18.pdf>
2. Public Health England. Update on investigation of UK case of Neisseria gonorrhoeae with high-level resistance to azithromycin and resistance to ceftriaxone acquired abroad. Health Protection Report. April 20, 2018. Link: <https://assets.publishing.service.gov.uk/government/uploads/system/uploads/attachment_data/file/701185/hpr1418_MDRGC.pdf>
3. Whittles LK, White PJ, Paul J, Didelot X. Epidemiological Trends of Antibiotic Resistant Gonorrhoea in the United Kingdom. Antibiotics (Basel). 2018 Jul 13;7(3). pii: E60. doi: 10.3390/antibiotics7030060. Review.
4. Semchenko EA, Tan A, Borrow R, Seib KL. The serogroup B meningococcal vaccine Bexsero elicits antibodies to Neisseria gonorrhoeae. Clin Infect Dis. 2018 Dec14. doi: 10.1093/cid/ciy1061. [Epub ahead of print]
5. Petousis-Harris H, Paynter J, Morgan J, Saxton P, McArdle B, Goodyear-Smith F, Black S. Effectiveness of a group B outer membrane vesicle meningococcal vaccine against gonorrhoea in New Zealand: a retrospective case-control study. Lancet. 2017 Sep 30;390(10102):1603-1610. doi: 10.1016/S0140-6736(17)31449-6. Epub 2017 Jul 10.
6. Humbert MV, Christodoulides M. Immunization with recombinant truncated Neisseria meningitidis-Macrophage Infectivity Potentiator (rT-Nm-MIP) protein induces murine antibodies that are cross-reactive and bactericidal for Neisseria gonorrhoeae. Vaccine. 2018 Jun 22;36(27):3926-3936. doi: 10.1016/j.vaccine.2018.05.069. Epub 2018 May 24.
7. Acevedo R, Bai X, Borrow R, Caugant DA, Carlos J, Ceyhan M, Christensen H, Climent Y, De Wals P, Dinleyici EC, Echaniz-Aviles G, Hakawi A, Kamiya H, Karachaliou A, Lucidarme J, Meiring S, Mironov K, Sáfadi MAP, Shao Z, Smith V, Steffen R, Stenmark B, Taha MK, Trotter C, Vázquez JA, Zhu B. The Global Meningococcal Initiative meeting on prevention of meningococcal disease worldwide: Epidemiology, surveillance, hypervirulent strains, antibiotic resistance and high-risk populations. Expert Rev Vaccines. 2019 Jan;18(1):15-30.
8. Public Health England. MenB vaccination: introduction from September 2015. Correspondence. June 2, 2015. Link: <https://www.gov.uk/government/publications/menb-vaccination-introduction-from-1-september-2015>
9. Christensen H, Trotter CL, Hickman M, Edmunds WJ. Re-evaluating cost effectiveness of universal meningitis vaccination (Bexsero) in England: modelling study. BMJ. 2014 Oct 9;349:g5725. doi: 10.1136/bmj.g5725.

**Appendix 1:** Data extraction form template

| **Study** | **Infections disease system** | **Model Type** | **Model formulation/ class** | **Transmission Route** | **Methodology used** | **Validation technique** | **Intervention**  **target** | **Type of data used** |
| --- | --- | --- | --- | --- | --- | --- | --- | --- |
| Author (year)  Country | Neisseria gonorrhoeae, Meningococcal Infections,  Antimicrobial Resistant | Population-based dynamic transmission model  Markov model  Economic model  Cohort model | Stochastic  Deterministic  Statistical  Hybrid  SIS  SVIR  SEIR  SIR  Age-structured  Multi-strain model | Oral, Co-infection, Sexual, | Differential equations (ODE or PDE);Jump processes (stochastic); Analytical; Neural networks | Model fitting:  Model Calibration: | Disease:  AMR:  Both:  N/A: | Clinical;  Epidemiological;  Experimental;  Theoretical |

**Appendix 2:**

| **Table 1: Search String** |
| --- |
| **Ovid MEDLINE(R) and Epub Ahead of Print, In-Process, In-Data-Review & Other Non-Indexed Citations, Daily and Versions(R) <1946 to June 28, 2021>** |
| **Communicable term**  1 (communicable or seroepidemiolog$ or transmit$ or transmission$).mp. = 776476  **Mathematical Model term**  2 Models, theoretical/ 156212  3 Markov Chains/ 15062  4 (compartmental adj3 model$).mp. 4786  5 micro simulation$.mp. 171  6 (mathematical adj3 model$).mp. 58146  7 2 or 3 or 4 or 5 or 6 219047  **Diseases term**  8 Gonorrhea/ 14370  9 Neisseria gonorrhoeae/ 10170  10 Meningococcal Vaccines/ 3616  11 Meningococcal Infections.mp. 6669  12 8 or 9 or 10 or 11 27746  **AMR & Multidrug**  13 Drug Resistance, Bacterial/ 43264  14 Antimicrobial Resistan*.mp. 27877  15 Bexsero.mp. 179  16 MenB.mp. 465  17 Drug Resistance, Multiple, Bacterial/ 22499  18 13 or 14 or 15 or 16 or 17 81675  **Results**  19 1 and 7 and 12 and 18 15  20 1 and 7 and 12 100 |

**Appendix 3. : SCOPUS**

(communicable or seroepidemiolog$ or transmit$ or transmission$) AND (Models, theoretical/        OR Markov Chains/         OR “compartmental model$” OR “micro simulation$”) AND (Gonorrhea/ OR Neisseria gonorrhoeae/ OR Meningococcal Vaccines/ OR Meningococcal Infections) AND (“Drug Resistance” OR Bacterial OR “Antimicrobial Resistan*” OR Bexsero OR MenB OR “Drug Resistance” OR ”multi-drug”)

**Appendix 4 : EMBASE**

| **Table 2: Search String** |
| --- |
| **Embase <1974 to 2021 July 27>** |
| **Communicable term**  1 (communicable or seroepidemiolog$ or transmit$ or transmission$).mp. 890080  **Mathematical Model term**  2 Models, theoretical/ 63104  3 Markov Chains/ 7179  4 (compartmental adj3 model$).mp. 6336  5 micro simulation$.mp. 340  6 (mathematical adj3 model$).mp. 158548  7 2 or 3 or 4 or 5 or 6 228967  **Diseases term**  8 Gonorrhea/ 17514  9 Neisseria gonorrhoeae/ 16251  10 Meningococcal Vaccines/ 7620  11 Meningococcal Infections.mp. 884  12 8 or 9 or 10 or 11 36803  **AMR & Multidrug**  13 Drug Resistance, Bacterial/ 151385  14 Antimicrobial Resistan*.mp. 34548  15 Bexsero.mp. 400  16 MenB.mp. 568  17 Drug Resistance, Multiple, Bacterial/ 45340  18 13 or 14 or 15 or 16 or 17 196366  **Results**  19 1 and 7 and 12 and 18= 33  20 1 and 7 and 12 =178 |
