# Supplementary material for: A systematic review to identify research gaps in studies modeling MenB vaccinations against Neisseria infections
Source: PLoS One. 2025 Jan 2;20(1):e0316184. doi: 10.1371/journal.pone.0316184 (PMC11694989; doi:10.1371/journal.pone.0316184)
Supplement: Attachment — Submitted filename: response.docx [file pone.0316184.s008.docx]

Editor’s comments

When submitting your revision, we need you to address these additional requirements.

1. Please ensure that your manuscript meets PLOS ONE's style requirements, including those for file naming. The PLOS ONE style templates can be found at 
https://journals.plos.org/plosone/s/file?id=wjVg/PLOSOne_formatting_sample_main_body.pdf and 
https://journals.plos.org/plosone/s/file?id=ba62/PLOSOne_formatting_sample_title_authors_affiliations.pdf

🡪 we have adjusted manuscript styles to PLOS One requirements, including SI file naming,

2. Please note that funding information should not appear in any section or other areas of your manuscript. We will only publish funding information present in the Funding Statement section of the online submission form. Please remove any funding-related text from the manuscript.

🡪 we have removed the funding statement from the manuscript.

3. Thank you for stating the following in the Competing Interests section: 
   "I have read the journal's policy and the authors of this manuscript have the following competing interests:
RB performs contract research on behalf of UKHSA for GSK, Pfizer, and Sanofi. RV has received research funding for PHE from GSK and Gilead Sciences in the past. All other authors have declared that no competing interests exist."

We note that you received funding from a commercial source: GSK and Gilead Sciences

Please provide an amended Competing Interests Statement that explicitly states this commercial funder, along with any other relevant declarations relating to employment, consultancy, patents, products in development, marketed products, etc.

Within this Competing Interests Statement, please confirm that this does not alter your adherence to all PLOS ONE policies on sharing data and materials by including the following statement: "This does not alter our adherence to PLOS ONE policies on sharing data and materials.” (as detailed online in our guide for authors http://journals.plos.org/plosone/s/competing-interests).  If there are restrictions on sharing of data and/or materials, please state these. Please note that we cannot proceed with consideration of your article until this information has been declared.

Please include your amended Competing Interests Statement within your cover letter. We will change the online submission form on your behalf.

🡪 The new funding statement reads:
“RB performs contract research on behalf of UKHSA for GSK, Pfizer, and Sanofi. RV has received research funding on behalf of Public Health England from GSK and Gilead Sciences in the past. This does not alter our adherence to PLOS ONE policies on sharing data and materials. All other authors have declared that no competing interests exist.”
We have included the statement in the cover letter.

4. We note that your Data Availability Statement is currently as follows: All relevant data are within the manuscript and its Supporting Information files.

Please confirm at this time whether or not your submission contains all raw data required to replicate the results of your study. Authors must share the “minimal data set” for their submission. PLOS defines the minimal data set to consist of the data required to replicate all study findings reported in the article, as well as related metadata and methods (https://journals.plos.org/plosone/s/data-availability#loc-minimal-data-set-definition).

For example, authors should submit the following data:

- The values behind the means, standard deviations and other measures reported;
- The values used to build graphs;
- The points extracted from images for analysis.

Authors do not need to submit their entire data set if only a portion of the data was used in the reported study.

If your submission does not contain these data, please either upload them as Supporting Information files or deposit them to a stable, public repository and provide us with the relevant URLs, DOIs, or accession numbers. For a list of recommended repositories, please see https://journals.plos.org/plosone/s/recommended-repositories.

If there are ethical or legal restrictions on sharing a de-identified data set, please explain them in detail (e.g., data contain potentially sensitive information, data are owned by a third-party organization, etc.) and who has imposed them (e.g., an ethics committee). Please also provide contact information for a data access committee, ethics committee, or other institutional body to which data requests may be sent. If data are owned by a third party, please indicate how others may request data access.

🡪 Our submission did contain all raw data except for the underlying numbers for Fig 2. We have now included these numbers in table format in Tab S3.

5. Please ensure that you refer to Figure 3 in your text as, if accepted, production will need this reference to link the reader to the figure.

🡪 Fig 3 is referred to in section 3.3.1.

6. Please include captions for your Supporting Information files at the end of your manuscript, and update any in-text citations to match accordingly. Please see our Supporting Information guidelines for more information: http://journals.plos.org/plosone/s/supporting-information.

🡪 We have included a section for SI at the end of the manuscript and updated in text citations accordingly.

7. Please review your reference list to ensure that it is complete and correct. If you have cited papers that have been retracted, please include the rationale for doing so in the manuscript text, or remove these references and replace them with relevant current references. Any changes to the reference list should be mentioned in the rebuttal letter that accompanies your revised manuscript. If you need to cite a retracted article, indicate the article’s retracted status in the References list and also include a citation and full reference for the retraction notice.

🡪 we have double checked all references to make sure they are correct, and none was retracted.


Reviewers' comments

5. Review Comments to the Author

Please use the space provided to explain your answers to the questions above. You may also include additional comments for the author, including concerns about dual publication, research ethics, or publication ethics. (Please upload your review as an attachment if it exceeds 20,000 characters)

Reviewer #1: This systematic review focuses on studies on vaccination strategies against Neisseria incidence and antimicrobial resistance. The primarily aim is to identify gaps in research and provide suggestions for future research, including the combination of both meningococcal and gonococcal infections in studies to provide better estimates for vaccine benefits. The review is well written and the topic is relevant.

🡪 We thank reviewer 1 for their comments.

I have a few minor comments below:
- Typo in Results section paragraph 3.2 where the sentence "Error! Reference source not found." is included.

🡪 corrected

- Paragraph 3.3.1: Could the authors provide a brief definition of difference between deterministic ODE and stochastic Markov models?

🡪 We have included the brief descriptions for the two model types:
“Ordinary differential equation (ODE) models are widely used for modelling dynamic transmission of diseases because they are simpler to implement and interpret with a single deterministic outcome. Markov models account for random variation in their inputs and yield outcome probabilities, thus illustrating the uncertainty in the process.”

- Table 2: there is a typo for the year of publication which is 2016 and not 2006

🡪 corrected

- Point 3 in the Discussion section: should be sequelae instead of sequalae?

🡪 corrected

- Figure 2 is not readable and same for Figure 3

🡪 we are unsure why this was the case, we have re-uploaded all figures, complying with PLOS One figure standards

Reviewer #2: This is a very interesting topic for a review. However the paper has major limitations.

🡪 We thank reviewer 2 for the comments.

First of all, the paper only focuses on Men B vaccination and its effect on N. gonorrhoeae. The authors should make the goal of their study more clear in the title and abstract, as they explicitly exclude all studies on meningococcal conjugate vaccine that are not type B.

🡪 The reviewer is correct in that we only focus on Men B vaccination (and its effect on both N. gonorrhoeae and N. meningitidis). We have now made this clear in title and abstract.

Second, the result section should include more details on the studies included in the review: what is the data used (did they use disease incidence records? were the studies mostly free simulations or did they provide inference based on real data?) , what was the impact of vaccination at the population level? The authors need to describe more in details these results for this review to be useful for researchers.

🡪 We agree with the reviewer’s suggestion that information on used data for model calibration and validation, treatment impact on AMR, and vaccine impact on the population would be a useful addition to the results section. Please see details added in sections 3.3.1, 3.3.3, 3.3.7, and 3.4.4 - 3.4.6.

While revising your submission, please upload your figure files to the Preflight Analysis and Conversion Engine (PACE) digital diagnostic tool, <https://pacev2.apexcovantage.com/>. PACE helps ensure that figures meet PLOS requirements. To use PACE, you must first register as a user. Registration is free. Then, login and navigate to the UPLOAD tab, where you will find detailed instructions on how to use the tool. If you encounter any issues or have any questions when using PACE, please email PLOS at figures@plos.org. Please note that Supporting Information files do not need this step.

🡪 we have used PACE for all our figures.
